# Peritoneal Dialysis as a First versus Second Option after Previous Haemodialysis: A Very Long-Term Assessment

**DOI:** 10.1155/2014/693670

**Published:** 2014-11-20

**Authors:** Roberto José Barone, María Inés Cámpora, Nélida Susana Gimenez, Liliana Ramirez, Sergio Alberto Panese, Mónica Santopietro

**Affiliations:** Peritoneal Dialysis Program, Hurlingham Renal Therapy Services, 1431 Buenos Aires, Argentina

## Abstract

For renal replacement therapy, overall survival is more important than the choice of currently available individual therapy. *Objectives*. To compare patients and technique survival on peritoneal dialysis as first treatment (PDF) versus after previous haemodialysis (HDPD) and other indicators of follow-up. *Methods*. We prospectively studied 110 incident patients, during the period from August 4, 1993, to June 30, 2012, for patients and technique survival (Kaplan-Meier) (log rank *P* < 0.05). *Results*. Groups: (A) PDF: 37 patients, 24 females, age: 52.2 ± 14.9 years old, time at risk: 2123 patient-months (p/m), mean: 57 ± 42 months; (B) HDPD: 73 patients, 42 females, age: 52.45 ± 14.7 years old, time in haemodialysis: 3569.2 (p/m), range: 3–216 months, mean: 49 ± 45 months, time at risk in PD: 3700 (p/m), mean: 51 ± 49 months. Patients' survival: (A) PDF: 100%, 76.6%, 65.6%, and 19.7%; (B) HDPD: 95.4%, 65.6%, 43%, and 43% at 12, 60, 120, and 144 months, respectively, *P* = 0.34. Technique: (A) PDF: 100%, 90%, 59.8%, and 24%; (B) HDPD: 94%, 75%, 32%, and 32% at 12, 60, 120, and 144 months, respectively, *P* = 0.40. *Conclusions*. Comparable patient and technique survival were observed. Peritoneal dialysis enables a greater extension of renal replacement therapy for patients with serious difficulties continuing with haemodialysis.

## 1. Introduction

Since the beginning of the chronic renal substitutive therapy, many advances have been made regarding medical and technological aspects that have undoubtedly contributed to the quality of life and survival of the patients [1–4]. Too much time was previously spent by nephrologists attempting to understand that the treatments that are currently available—haemodialysis, peritoneal dialysis, and renal transplantation—are part of a group of options enabling patients to live their life; the challenge for the nephrologist must be continuing with advances in the replacement therapy area and renal prevention avoiding meaningless confrontations among them concerning the treatments [5, 6].

From the early years of the peritoneal dialysis, countless articles have been published comparing peritoneal dialysis versus haemodialysis in many aspects of the replacement therapy like anaemia, adequacy, residual renal function impact, quality of life, patient satisfaction, cost of treatment, reimbursement, and so on, but patient survival is undoubtedly the most important index that stresses the effectiveness of a therapy. Likewise, in this aspect, technique survival is another important indicator that is often cited as information that is “required” of the peritoneal dialysis performance; however, papers showing technique survival in haemodialysis are lacking.

In PD, comparisons among diabetic and nondiabetic and anuric patients and patients with residual renal function are frequent, but comparisons between patients undergoing PD as first option versus PD as a second option after haemodialysis are scarce [7–10].

In this work, we showed our experience in very long-term treatment, comparing technique, catheter, and patient survival, as well as other indices of those patients who initiated peritoneal dialysis as a first option versus patients who initiated substitutive therapy in haemodialysis and were transferred to peritoneal dialysis as a second option for different reasons.

## 2. Methods

We prospectively evaluated 110 patients who had been undergoing continuous ambulatory peritoneal dialysis (CAPD) or automated PD (APD) for at least three months during the period August 4, 1993, to June 30, 2012. We established two groups: (A) all incident patients who initiated renal substitutive therapy in peritoneal dialysis as a first option (PDF) and (B) patients who were first treated in haemodialysis for more than three months and then switched to PD as a second option (HDPD) for different reasons. Patients with 24 hr urine volumes at the start of PD lower than 100 mL/day were considered anuric.

We used the Kaplan-Meier product-limit estimation method to calculate patients and technique survival as “intention-to-treat survival” and catheter survival. For patients' survival, death was considered the endpoint. For technique survival, transfer to haemodialysis or death related to PD therapy was considered the endpoint. Patients who were transplanted or lost to follow-up or who achieved partial recovery of renal function were censored. Furthermore, patients and technique survivals between PDF patients and anuric patients of the HDPD group were compared. For catheter survival, catheter removal was considered the endpoint; patients whose only reason for catheter extraction was transplantation, elective transfer to haemodialysis, or death from concurrent disease with functioning catheter were censored at time of that event.

The Kaplan-Meier curve comparisons mentioned previously between both groups of patients were performed using the log-rank method. Cumulative peritonitis rate (CPR) in both groups was measured.

Overall admission rates and hospital days per patient year per group were calculated and compared (unpaired* t*-test) during the study period. In our peritoneal dialysis program, the patients were not hospitalised for training in dialysis. Hospitalisation for the first peritoneal catheter placement was not considered in the morbidity evaluation. The chi-square test was used to analyse the proportion of patients hospitalised per group and number of diabetic patients per group. Relative risk (RR) for mortality was used to determine the impact of diabetes as morbid risk factor.

Adequacy studies were performed every 3 to 6 months; weekly total urea clearance (Kt/V) and weekly total creatinine clearance/1.73 m² were calculated. We measured total body water (TBW) according to the Watson formulas [11] and body surface area (BSA) was calculated according to D. D. Bois and E. F. D. Bois [12].

 For women,  TBW = −2.097 + (0.1069 · Height)+(0.2466 · Weight).
 For men,  TBW = 2.447 − (0.09156 · Age) + (0.1074 · Height) + (0.3362 · Weight).
 BSA (m^2^) = 0.007184 *·* Height (cm) 0.725 *·* Weight (kg) 0.425.

The normalized protein catabolic rate (nPCR) was calculated by the Randerson formula:

 nPCR = 10.76 (Gun + 1.46)/*V*, where Gun is urea nitrogen generation rate (mg/min) and *V* is volume of urea distribution [13].

Mean comparisons of the following indices were performed during the study period (unpaired *t*-test): weekly Kt/V, total weekly creatinine clearance, weekly peritoneal urea clearance, weekly renal urea clearance, BSA, TBW, nPCR, total daily drainage volume, and total daily drainage volume/m² BSA. The data were collected prospectively from our database. Continuous variables are expressed as mean ± standard deviation; categorical data are expressed as frequencies and percentages. A *P* value of 0.05 or less was considered statistically significant. The statistical analysis was performed with SPSS 15 (SPSS Inc., Chicago, IL, EEUU).

## 3. Results

Our study enrolled 110 patients, who were divided into two groups: (A) PDF: there were 37 patients, 24 females (64.8%) and 13 males; the mean age was 52.2 ± 14.9 years old and the age range when they started PD was 16–75 years; the time at risk was 2123 patient-months, mean: 57 ± 42 months; 14 patients (37.84%) switched from CAPD to APD; the percentage of diabetic patients was 27% and the cumulative peritonitis rate was 0.34, (B) HDPD: it included 73 patients, 42 females (57.53%) and 31 males; mean age was 52.45 ± 14.7 years old and the age range when patients initiated PD was 22–80 years; the time at risk in PD was 3700 patient-months, mean: 51 ± 49 months; 33 patients (45%) switched from CAPD to APD; the percentage of diabetic patients was 15%; 48 patients (65.7%) were anuric and the cumulative peritonitis rate was 0.36. In the latter group of patients, the time at risk in haemodialysis was 3569.2 patient-months, mean: 49 ± 45 months and range: 3 to 216 months. Furthermore, in this group, 43 patients (58.9%) were shifted from haemodialysis to peritoneal dialysis due to multiple vascular access failure; 32 (74.4%) of these patients were women; 32.8% switched by personal choice; 6.85% switched due to cardiovascular disorders and 1.37% due to living a long distance from the dialysis centre. No statistical significance was observed in the number of diabetic patients per group (*P* = 0.21). Patients' characteristics are shown in Table 1.

It is important to point out that 35 (47.9%) out of 73 patients in the second group moved onto our peritoneal dialysis program from other dialysis clinics because peritoneal dialysis was not practiced in those units.

**Figure 1:**
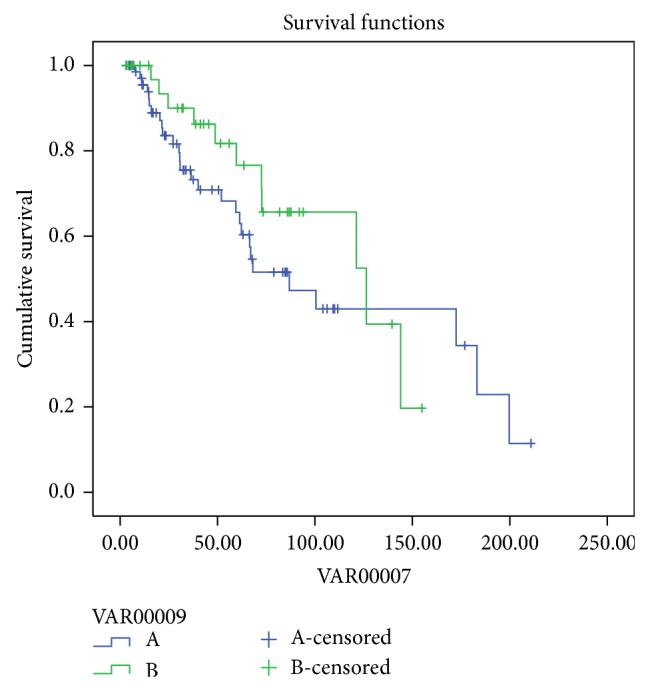
Patients survival PDF (green) and HDPD (blue) (log rank *P* = 0.33).

The probability of patient's survival in group (A) PDF at 12, 36, 60, 84, 120, and 144 months was 100%, 90%, 76.6%, 65.6%, 65.6%, and 19.7%, respectively, and, in group (B) HDPD, at 12, 36, 60, 84, 120, 144, and 180 months, it was 95.4%, 75.5%, 65.6%, 51.5%, 43%, 43%, and 34%, respectively (log rank *P* = 0.33) (Figure 1).

**Figure 2:**
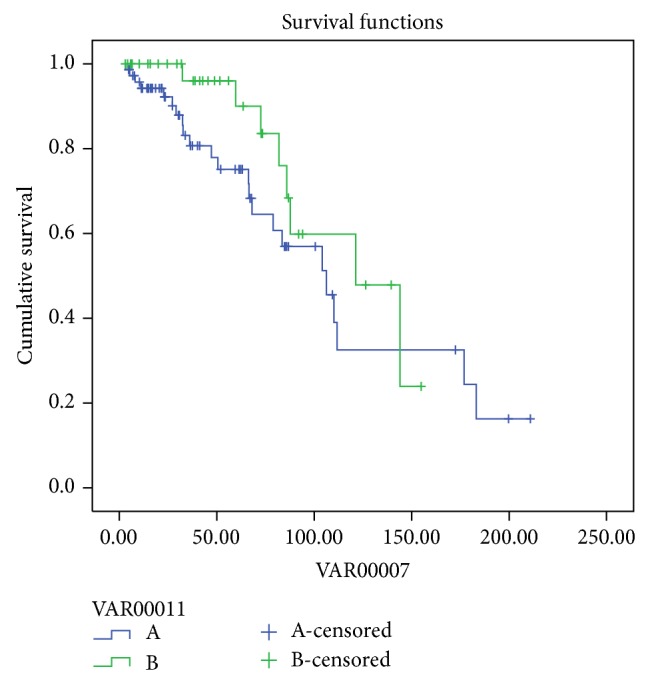
Technique survival PDF (green) and HDPD (blue) (log rank *P* = 0.20).

The estimation of technique survival was 100%, 96%, 90%, 76%, 59.8%, and 24% at 12, 36, 60, 84, 120, and 144 months, respectively, in group A, and 94%, 83%, 75%, 57%, 32%, 32%, and 24% at 12, 36, 60, 84, 120, 144, and 180 months, respectively, in group B (log rank *P* = 0.20) (Figure 2).

**Figure 3:**
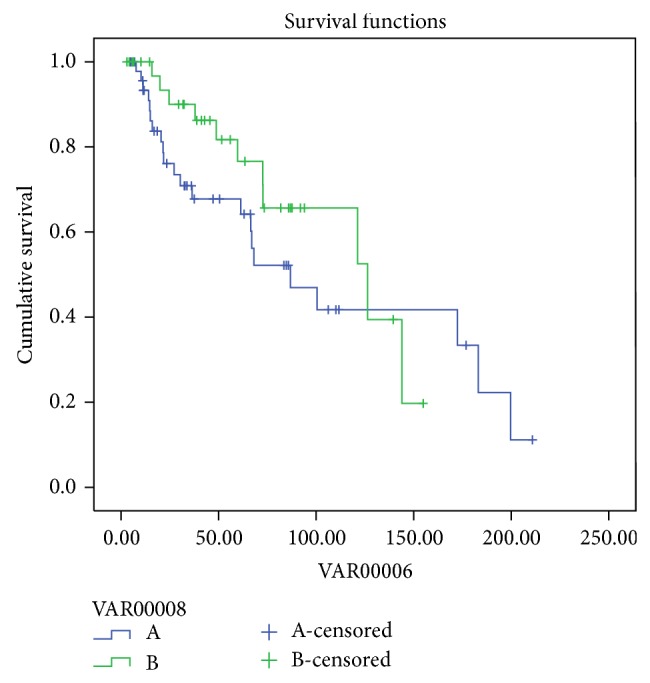
Patients survival PDF (green) and HDPD (anuric) (blue) (log rank *P* = 0.31).

**Figure 4:**
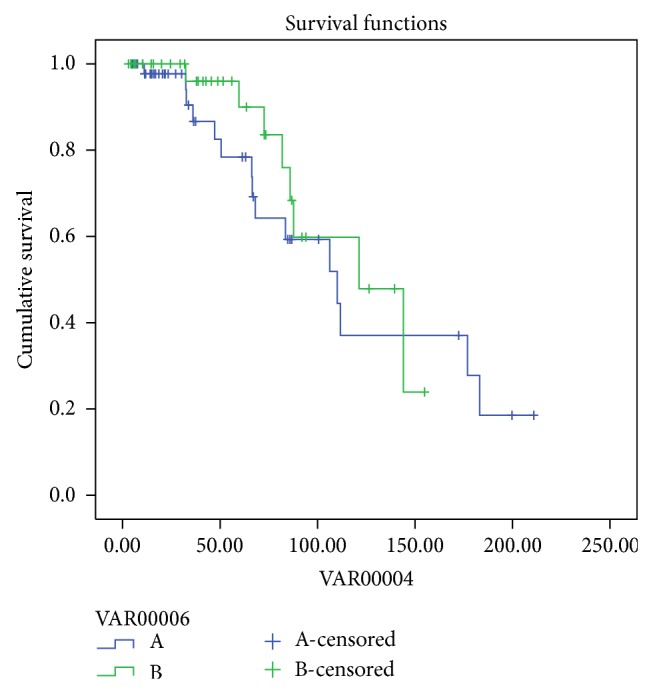
Technique survival PDF (green) and HDPD (anuric) (blue) (log rank *P* = 0.48).

No statistical significance was observed when patient and technique survival were compared between patients of group A (all patients started PD with residual renal function) and the anuric patients of the HDPD group (log rank *P* = 0.31 and *P* = 0.48, resp.) (Figures 3 and 4).

**Figure 5:**
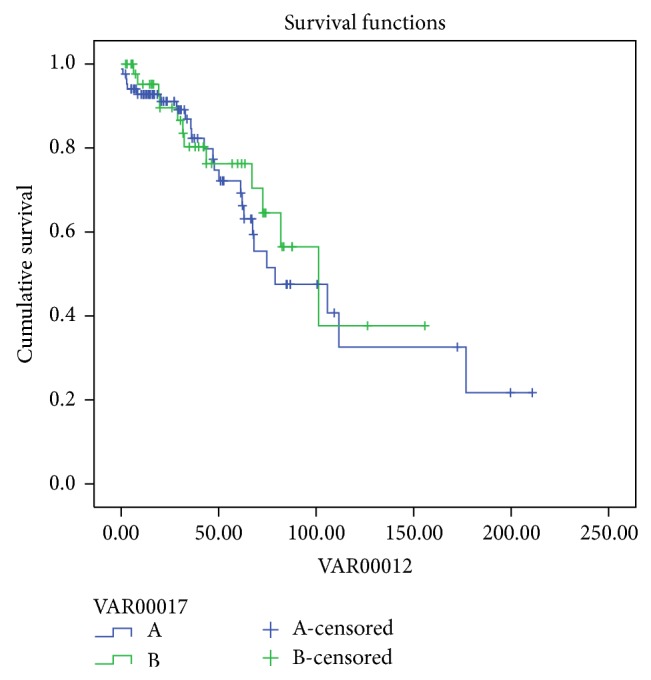
Catheters survival PDF (green) and HDPD (blue) (log rank *P* = 0.62).

Forty-seven catheters (35 swan neck and 12 Tenckhöff ones) were placed in the PDF group (1.27 catheters per patient) and 85 (62 swan neck and 23 Tenckhöff) in the HDPD group (1.16 catheters per patient) during the period of study. The observation of the catheters survival in group A at 12, 36, 60, 84, and 144 months was 95%, 80%, 76%, 56%, and 37%, respectively, and in group B, at 12, 36, 60, 84, 144, and 180 months, it was 93%, 84%, 72%, 47%, 32%, and 22%, respectively (log rank *P* = 0.62) (Figure 5).

Unadjusted hospitalisation rates were similar for the groups. During the study period there were forty-five admissions (16% of cardiovascular cause, 26.6% due to peritonitis) in 21 out of 37 patients of the PDF group, which equates to 0.25 admissions per patient/year, and the numbers of hospital days were 1.95 per patient/year. In the HDPD group, there were 98 admissions (27.5% of them due to cardiovascular disorders and 17.5% for peritonitis) in forty-nine out of 73 patients in the time at risk, which equates to 0.32 admissions per patient/year, and the number of days of hospitalisation per patient/year was 1.88. There were no statistical differences in the proportion of patients hospitalised or the proportion of admissions due to cardiovascular disorders (*P* = 0.28 and *P* = 0.29, resp.), in neither the number of admissions nor the duration of hospitalisations (unpaired *t*-test) (*P* = 0.55 and *P* = 0.62, resp.). Furthermore, the number of admissions per patient/year for peritonitis was 0.06 for the first group and 0.07 for HDPD patients (*P* = 0.41).

The RR for diabetes was 0.60 and 0.67 in the PDF and HDPD groups, respectively. Body surface area and total body water were comparable between the groups; the observation of the mean adequacy indices (Kt/V and total weekly creatinine clearance) and nPCR did not show statistical significance, but the mean values of peritoneal and renal urea clearances and total daily drainage volume were statistically significant (Table 2).

## 4. Discussion

There are innumerable publications that have shown the attributes of the peritoneal dialysis as substitutive renal therapy. In the decades of the eighties and nineties, researchers published comparisons between peritoneal dialysis and haemodialysis regarding patient and technique survival with dissimilar results [14–34]. Nowadays, patient survival and the relationship between modality of dialysis and mortality are an unsolved debate. van Biesen and coworkers introduced the concept of integrative care of end stage renal disease patients using both modalities of treatment according to individual needs [35, 36]. In the last few years, the concept “PD first” has deeply impacted the nephrologists' circle; however, this impact was not translated into the growth of this treatment worldwide [37]. Chaudhary et al., among others, described the advantages of peritoneal dialysis as the first modality and the reasons of underutilisation [14]. Some studies show the outcome of patients transferred from peritoneal dialysis to haemodialysis, but long-term studies analysing the outcome of patients transferred from haemodialysis to peritoneal dialysis are sparse [7–10].

In Argentina, the relation haemodialysis/peritoneal dialysis patients is about 96%/4%, respectively; thus, peritoneal dialysis population is too scarce. Our paper shows comparisons of some of the most important indices of follow-up of patients undergoing peritoneal dialysis on the very long term, with those patients who initiated peritoneal dialysis as first option compared to those transferred from haemodialysis to peritoneal dialysis. We believed that the sample of patients in this aspect is acceptable due to such a long time of follow-up. It is important to point out that many patients moved from other clinics to our peritoneal dialysis program after having been exposed to between two and thirty-three procedures of vascular access among native and prosthetic fistulas in arms and transitory or permanents catheters in veins subclavia, jugular and femoral before starting peritoneal dialysis.

Patient and technique survival are some of the most important indices in the assessment of the substitutive therapies; in our study, the comparison between both groups did not show statistical differences. Similar findings were observed by Zhang and coworkers [10]. Residual renal function plays an important role in the solute clearance and in fluids balance in the dialysis population; Heaf et al. inferred that preservation of the RRF in peritoneal dialysis could be a cause of better survival in the first 2 years of dialysis treatment regarding HD [38]. Some studies support that the diminution of urine volume is a predictor of technique failure and a cause of mortality [39–42]. In contrast, in the NECOSAD study, the authors considered anuric peritoneal dialysis patients to have acceptable patient and technique survival, and the risk factors for death were the same as in the dialysis population as a whole [43]; similar conclusions were found in the EAPOS study in anuric patients on APD [44] and in anuric patients with high body surface area [45]. Lobo and coworkers performed a nationwide study of 739 patients but did not find any differences between survival rates between patients with and patients without previous haemodialysis or in anuric or residual renal function patients [46]. As expected in our study, many patients from the second group were anuric (65.7%); however, we also did not observe statistical differences on the very long term when comparing these anuric patients versusPDF patients (Figures 3 and 4).

Residual renal function contributes to achieving adequacy targets; however, due to the fact that diuresis declines during the course of treatment, the dialysis prescription must be modified to maintain the adequacy level, especially for patients with high body weight [45, 47]. In our study, targets of small solute clearance were achieved in both groups of patients [48]. Although the participation of RRF is obvious in the first group, in the HDPD group, the target was reached relying heavily on peritoneal clearance, with the largest peritoneal fluid delivery as well; this was also observed by Bammens et al. [49] (Table 2). Many patients who started CAPD in both groups were transferred to APD to improve adequacy levels and meet target recommendations through optimisation of the transport characteristics of peritoneal membrane or to increase the UF volume. On the other hand, other reasons for transferring to APD were often linked to social situations, job or study possibilities, the needs of a partner, lifestyle, back pain, and so on.

A twenty-four-hour daily volume of ultrafiltration is very important in order to satisfy the individual negative daily balance requirements; Ateş et al. warned about the importance of the total fluid removal in the survival of the patients [50]. On the other hand, in many cases in which patients start PD as the first therapy, their urine output is important; if their blood pressure is under control, it is probable that it was not initially essential to get a negative fluid balance in excess of individual necessities; thus high glucose concentration solutions could be avoided. In our study, statistical differences observed between the groups regarding total daily drainage volume are linked to a smaller peritoneal volume prescribed at the beginning of PD due to the contribution of the RRF, which was mainly seen in the first group. The condition of diabetes surprisingly did not have impact as a risk factor of mortality in both groups of patients; nevertheless, there is an atypical prevalence of diabetic and nondiabetic patients' relation and its impact as risk factor observed in our study, in this distribution of patients, might have a bias because the sample was not taken at random.

Technique survival often depends on catheter-related problems; our catheter survival evaluation showed a satisfactory outcome in such a long-term follow-up regarding published data [51–53].

Hospitalisation is a very important indicator of morbidity in assessment of the peritoneal dialysis program, such as in haemodialysis. There are many publications comparing hospitalisations between PD and HD, but there are few and small studies in this aspect between patients who started PD as a substitutive therapy and patients treated sequentially with haemodialysis and peritoneal dialysis [54–56]. In our study period, we did not observe any statistical differences in admissions nor number of hospitalizations days per patients/year between both groups. Moreover, the results showed an overall low admission rate and very low rate regarding admissions for peritonitis [57, 58].

## 5. Conclusion

The outcome on the very long term of a medical therapy, in a way, discloses its effectiveness; the assessment of the recognised indicators for the replacement therapies of the end stage renal disease patients observed in our study shows that peritoneal dialysis as a first option and continuing haemodialysis are both highly reliable; also, the concept of integrative care is clear, allowing the life of patients to be prolonged. Therefore, it would be very positive to avoid risky vascular access procedures in excess for patients in conditions requiring peritoneal dialysis.

## Figures and Tables

**Table 1:** Patients characteristic.

Variables	Group A-PDF (*n* = 37)	Group B-HDPD (*n* = 73)
Age (years)	52.2 ± 14.9	52.45 ± 14.7
Age range (years)	16–75	22–80
Sex (male-female)	13–24	31–42
Time at risk on PD (p/m)	2123 57 ± 42	3700 51 ± 49
Time at risk on hemodialysis (p/m)	—	3569.2 49 ± 45
Diabetes (*n*)	10 (27%)	11 (15%)
Peritonitis rate	0.34	0.36

p/m: patient-months. Data are expressed as mean ± SD.

**Table 2:** Adequacy indices.

Variables	PDF	HDPD	*P* value
Total Kt/V (week)	2.26 ± 0.44	2.24 ± 0.56	NS
Total C. Cr (week)	71.97 ± 25.41	62.35 ± 18.7	NS
TBW	33.07 ± 6.91	33.8 ± 5.74	NS
BSA	1.68 ± 0.2	1.71 ± 0.17	NS
Peritoneal urea clearance (week)	54.83 ± 13.7	66.03 ± 11.79	*P* < 0.05
Renal urea clearance (week)	19.17 ± 18.7	6.39 ± 10.9	*P* < 0.05
nPCR	1.03 ± 0.2	1.06 ± 0.26	NS
Total daily drainage volume	8.98 ± 2.4	10.95 ± 1.67	*P* < 0.05
Total daily drainage volume/m² BSA	5.34 ± 1.23	6.42 ± 0.94	*P* < 0.05

Total body water (TBW), body surface area (BSA), and normalised protein catabolic rate (nPCR). Data are expressed as mean ± SD.
